# Investigation on the relationship between renal NONO expression, fibrosis and prognosis in diabetic nephropathy

**DOI:** 10.3389/fendo.2025.1652391

**Published:** 2025-10-14

**Authors:** Shuang Yang, Huiyuan Liao, Hanlu Ding, Xingli Xu

**Affiliations:** ^1^ Department of Nephrology, Sichuan Provincial People’s Hospital, Sichuan Clinical Research Center for Kidney Diseases, Clinical Immunology Translational Medicine Key Laboratory of Sichuan Province, University of Electronic Science and Technology of China, Chengdu, Sichuan, China; ^2^ Institute of Cardiovascular Diseases & Department of Cardiology, Sichuan Provincial People’s Hospital, School of Medicine, University of Electronic Science and Technology of China, Chengdu, Sichuan, China; ^3^ Department of Nephrology, The First Affiliated Hospital of Chengdu Medical College, Chengdu, China; ^4^ School of Clinical Medicine, Chengdu Medical College, Chengdu, China

**Keywords:** diabetic nephropathy, renal interstitial fibrosis, non-POU domain containing octamer-binding protein, matrix metalloproteinase-9, prognosis

## Abstract

**Background:**

Renal interstitial fibrosis (RIF) is an important manifestation of Diabetic nephropathy (DN) progression. Non-POU domain containing octamer-binding protein (NONO) is crucial in fibrosis in cardiovascular diseases, but its role in DN fibrosis remains unclear. This study explores the expression of NONO in DN and its correlation with Matrix Metalloproteinase-9 (MMP-9, as an important regulator of fibrosis), renal fibrosis, and prognosis.

**Methods:**

Forty patients with type 2 diabetes mellitus (T2DM) with pathologically confirmed DN were included, divided into early DN group (n=20) and late DN group (n=20). 6 normal renal tissue as control group. HE, Masson staining, immunohistochemical staining and Immunofluorescence double staining were performed. The correlation between NONO expression levels and MMP-9 as well as clinical pathological data was analyzed. Cox regression analysis and Kaplan-Meier survival curves were used to evaluate the relationship between renal tissue NONO expression levels and DN prognosis.

**Results:**

Compared with control group, NONO expression levels in renal tissues of DN patient were increased, and the late DN group was higher than the early DN group (*P*<0.05). NONO and MMP-9 expression were positively correlated with multiple clinical and Fibrosis-related pathological indicators, and NONO expression was positively correlated with MMP-9(*P*<0.05). Patients with high renal NONO expression had lower kidney progression-free survival rates.

**Conclusions:**

NONO expression levels correlate positively with MMP-9, collagen and renal damage indicators in renal tissues of DN patients. High NONO expression is linked to poor renal prognosis in DN. NONO may contribute to renal tissue fibrosis in DN by regulating MMP-9 levels.

## Graphical Abstract

We investigated the expression of the NONO protein across various stages of diabetic nephropathy and its association with clinical prognosis through renal tissue staining and statistical analysis, indicating that NONO may play a significant role in the pathogenesis and progression of diabetic nephropathy.

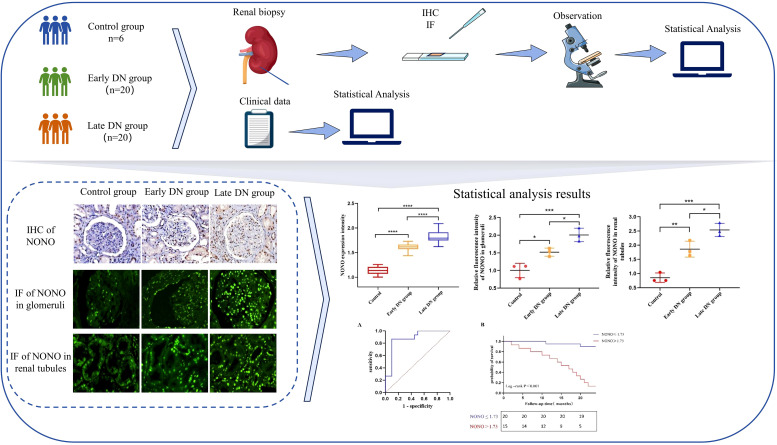

## 1 Introduction

Diabetic nephropathy (DN) is one of the most severe microvascular complications of diabetes mellitus (DM) and has become a leading cause of chronic kidney disease (CKD) and end-stage renal disease (ESRD) worldwide (1). Epidemiological studies show that approximately 30-50% of cases of ESRD are caused by DN. Epidemiological studies have shown that about 30-50% of ESRD cases are caused by DN, which has become one of the leading causes of death in patients with DM. With the increasing prevalence of DN, it also brings great health threat and economic burden to patients (1, 2). The typical clinical manifestations of DN are progressive proteinuria and renal function decline, with the disease process being insidious and easily missed in its early stages, potentially leading to renal failure (3). The main pathological features of DN include glomerular basement membrane thickening, glomerulosclerosis, tubular atrophy, renal interstitial fibrosis (RIF), and capillary damage (4). The primary pathological characteristic of RIF is epithelial-mesenchymal transition (EMT) of renal tubular epithelial cells, excessive deposition of extracellular matrix (ECM) in the renal interstitium, which leads to the replacement of normal tissue with fibrotic tissue, ultimately resulting in progressive loss of kidney function. Currently, the exact mechanism of RIF remains unclear, which increases the difficulty of antifibrotic treatment. Clinical management primarily involves controlling blood glucose, blood pressure, and lipid levels, while targeting multiple pathways to inhibit renal fibrosis. However, therapeutic outcomes remain suboptimal, and a significant number of patients ultimately progress to ESRD. Key pharmacological interventions include renin-angiotensin-aldosterone system inhibitors, sodium-glucose cotransporter 2 inhibitors, non-steroidal mineralocorticoid receptor antagonists, and glucagon-like peptide-1 receptor agonists, among others (5). An increasing number of studies suggest that RIF is a crucial factor in the progression of DN to ESRD and a reliable indicator for evaluating renal function prognosis. Therefore, in-depth research into the pathogenesis of RIF in DN is of great significance.

Non-POU domain containing octamer-binding protein (NONO) is a 54 kDa nuclear protein belonging to the Drosophila behavior/human splicing (DBHS) family, which is widely present in most mammalian cells (6). Recent studies have shown that NONO plays an important role in the fibrosis process of cardiovascular diseases (7). Ren et al. reported that the expression level of NONO was reduced in vascular smooth muscle cells and adventitial fibroblasts from patients with aortic dissection, and was significantly associated with collagen deposition and the degree of fibrosis (7). Another study suggested that NONO may participate in cardiac fibrosis in mice by targeting fibroblast proliferation, migration, and ECM deposition (8). This indicates that NONO plays a role in collagen formation and tissue fibrosis. Some studies have found that high expression of NONO protein can interact with NF-κB to promote the expression of matrix metalloproteinases-2 (MMP-2), matrix metalloproteinases-9 (MMP-9), and inflammatory cytokines, reducing the content of ECM, thereby participating in the regulation of atherosclerotic plaque stability in mice (10). The silencing of NONO can promote collagen formation and inhibit collagen degradation by upregulating P4Hα1 and downregulating MMP-2 and MMP-9 expression, thereby delaying the progression of abdominal aortic aneurysms (9). MMP-9 is a zinc-dependent endopeptidase that is a major physiological regulator of ECM degradation and also plays a key role in inducing EMT, inflammation, and fibrosis in kidney diseases (11, 12). Numerous studies have shown that MMP-9 is a key pathological mediator in renal fibrosis of DN, involved in the degradation of ECM components such as type I collagen (Col-I), and type III collagen (Col-III), disrupting the dynamic balance of ECM degradation and deposition, thus regulating the progression of fibrosis (13). However, the role of NONO in renal fibrosis has not been reported. Therefore, we hypothesize that NONO may mediate ECM expression by regulating MMP-9 levels, thereby participating in renal fibrosis.

In this study, we compare the expression levels of NONO, MMP-9, Col-I, and Col-III in kidney tissues from DN patients at different pathological stages, and analyze the data in combination with clinical biochemical indicators. Finally, Cox regression analysis and Kaplan-Meier survival curves are used to assess the relationship between the expression level of NONO in renal tissues and the prognosis of DN, further exploring the relationship between NONO and diabetic renal fibrosis. This research aims to provide new insights and evidence for the diagnosis and treatment of DN.

## 2 Materials and methods

### 2.1 Study design and patients

This study collected hospitalized patients diagnosed with type 2 DN through pathological biopsy at Sichuan Provincial People’s Hospital, Department of Nephrology, from January 2014 to December 2022 (40 cases) as the experimental group. The inclusion criteria followed the diagnostic standards for T2DM established by the American Diabetes Association and the diagnostic criteria for DN set by the Kidney Disease Outcomes Quality Initiative (14). Based on pathological biopsy staging, DN was classified into early-stage DN (stages I and II, n=20) and late-stage DN (stages III and IV, n=20) (15). Additionally, 6 patients with benign renal tumors were selected as the normal control group, with adjacent kidney tissue confirmed by renal biopsy to be free from abnormalities. The exclusion criteria were: (1) age <18 years; (2) non-type 2 diabetes patients; (3) patients with other primary or secondary kidney diseases; (4) patients with severe acute or chronic infections, malignant tumors, cardiovascular, cerebrovascular, immune, hematological, or other systemic diseases; (5) patients with acute diabetic complications or pregnancy; (6) patients who had undergone dialysis treatment. This study was approved by the Ethics Committee of Sichuan Provincial People’s Hospital (No. Lun Shen (Yan) 2024, No. 59) and informed consent was obtained from all enrolled subjects.

### 2.2 Data acquisition

We collected baseline clinical data, including gender (male/female), age (years), body mass index (BMI, kg/m^2^), duration of type 2 diabetes (years), history of hypertension (yes/no), systolic blood pressure (SBP, mmHg), and diastolic blood pressure (DBP, mmHg) measured at admission. Laboratory data included serum creatinine (Scr, umol/L), serum uric acid (SUA, umol/L), estimated glomerular filtration rate (eGFR, mL/min/1.73m^2^), blood urea nitrogen (BUN, mmol/L), serum albumin (Alb, g/L), hemoglobin (Hb, g/L), total cholesterol (TC, mmol/L), triglycerides (TG, mmol/L), high-density lipoprotein cholesterol (HDL-C, mmol/L), low-density lipoprotein cholesterol (LDL-C, mmol/L), fibrinogen (Fib, g/L), glycated hemoglobin (HbA1c, %), fasting blood glucose (FBG, mmol/L), urinary albumin/creatinine ratio (UACR, ug/mg), and 24-hour urinary protein (24hUpro, g/24h). All values were measured by routine clinical laboratories following standard procedures.

### 2.3 Histopathological analysis

Renal biopsy specimens from DN patients were collected to assess the proportion of interstitial fibrosis and renal tubular atrophy (IFTA) and the degree of inflammatory cell infiltration (evaluated by at least two nephropathologists). Hematoxylin-eosin (HE) staining was used to observe and assess renal tissue pathological damage (evaluated by at least two nephropathologists), and Masson staining was used to stain collagen fibers. Through microscopic observation, multiple random fields were selected, and the percentage of collagen-positive area relative to the total tissue area was analyzed and calculated using ImageJ software (16).

### 2.4 Immunohistochemistry

Renal tissue specimens were sectioned, dewaxed, hydrated, and subjected to antigen retrieval. After endogenous peroxidase (A8850, Solarbio, Beijing, China) blocking for 30 minutes, appropriate primary antibodies NONO antibody(ab50411, abcam, Cambridge, UK, 1:200,), MMP-9 antibody (A2095, abclonal, Wuhan, China, 1:200), Col-I antibody (ab34710, abcam, Cambridge, UK, 1:200), or Col-III antibody (ab7778, abcam, Cambridge, UK, 1:200) were added and incubated overnight at 4°C in a humidified box. The next day, an appropriate secondary antibody goat anti-mouse/rabbit IgG polymer (RS0011, Immunoway, California, USA) was added and incubated at room temperature for 20 minutes. After DAB (ZLI-9017, ZSGB-Bio, Beijing, China) color development, the sections were mounted with neutral balsam. The samples were observed under a microscope (DP73, Olympus, Tokyo, Japan), and multiple fields were randomly captured. Image J software was used to analyze and calculate the percentage of positive staining area in the total tissue area (16).

### 2.5 Immunofluorescence

Renal tissue sections were dewaxed, hydrated, and processed for antigen retrieval as in immunohistochemistry. After blocking with BSA (A8850, Solarbio, Beijing, China) for 30 minutes, NONO antibody (ab50411, abcam, Cambridge, UK, 1:100) and APQ1 (A15030, abclonal, Wuhan, China, 1:100) antibody or WT-1 antibody (A16298, abclonal, Wuhan, China, 1:100) diluted in PBST were added and incubated overnight at 4°C in a humidified box. The next day, secondary antibodies Alexa Fluor 488 (A32814, Jackson ImmunoResearch, Pennsylvania, USA)-labeled NONO antibody, Alexa Fluor 594 (A32754, Jackson ImmunoResearch, Pennsylvania, USA)-labeled APQ1 or WT-1 antibody were added (dilution 1:500) and incubated at room temperature for 1 hour in the dark. After DAPI (ab104139, Abcam, Cambridge, UK) staining of the nuclei, the sections were mounted with anti-fade mounting medium. Fluorescent images were collected using a confocal fluorescence microscope (LSM710, Leica, Wetzlar, Germany), and Image J software was used to analyze and calculate the average fluorescence intensity of NONO expression (17).

### 2.6 Kidney outcomes

The kidney outcome time was defined as the occurrence of a 50% reduction in eGFR from baseline or the initiation of renal replacement therapy (hemodialysis, peritoneal dialysis, or kidney transplantation) within 24 months of follow-up (18). Outpatient and inpatient case systems were used for follow-up tracking, and for patients not regularly followed up at our hospital, follow-up data were obtained through phone interviews. The time from enrollment to the occurrence of a renal endpoint event was recorded as the kidney progression-free survival time, measured in months. Patients who experienced renal endpoint events during follow-up were categorized into the poor prognosis group. The remaining patients were divided into the good prognosis group (n=20) and the poor prognosis group (n=15). Five patients lost to follow-up were excluded.

### 2.7 Statistical analysis

SPSS statistical software was used for all data analysis. All clinical and pathological data were tested for normality. Data conforming to a normal distribution are expressed as mean ± standard deviation (Mean ± SD), while non-normally distributed data are expressed as median and interquartile range (M (QL, QU)). Categorical data are expressed as percentages. For data that followed a normal distribution, independent sample t-tests or one-way analysis of variance (ANOVA) were used for inter-group comparisons. For categorical variables, the χ² test or Fisher’s exact test was used for comparison between groups. Non-normally distributed data were analyzed by the rank-sum test. Pearson correlation analysis was used for normally distributed quantitative data, and Spearman correlation analysis was used for non-normally distributed quantitative data, with correlation coefficients (r) and p-values reported. Multivariate linear regression analysis was performed to evaluate independent influencing factors of NONO expression in DN. Cox regression analysis was used to investigate the relationship between renal tissue NONO expression and renal prognosis. ROC curves were used to assess the ability of renal tissue NONO expression to distinguish patients with poor renal prognosis. Kaplan-Meier survival analysis was used to evaluate the relationship between NONO expression levels and DN prognosis. Image J software was used for quantitative analysis of pathological staining (Masson staining, immunohistochemistry, immunofluorescence). Statistical charts were plotted using GraphPad Prism 8.0.2. A *p*-value of <0.05 was considered statistically significant.

## 3 Results

### 3.1 Comparison of general data and laboratory indicators of all DN patients

A total of 40 patients were enrolled in this study, with 20 in the early DN group and 20 in the late DN group. Among them, 28 were male (70%) and 12 were female (30%), with an average age of 54.53 ± 8.47 years and an average BMI of 25.36 ± 3.30 kg/m². We analyzed and compared the differences in general data and research indicators between the two groups. The results showed significant statistical differences between the early and late DN groups in gender, UACR, 24hUpro, BUN, Scr, eGFR, Hb, Alb, Fib, TC, HDL-C, the proportion of IFTA involvement, and the degree of inflammatory cell infiltration (*P*<0.05). However, there were no statistically significant differences between the two groups in terms of age, BMI, duration of diabetes, history of hypertension, SBP, DBP, SUA, HbA1c, FBG, TG, and LDL-C (*P* > 0.05) (**Table 1**).

**Table 1:** Comparison of general information and laboratory indicators of patients.

Characteristic	Total (n=40)	Early DN group (n=20)	Late DN group (n=20)	t/χ2/Z	*P*-value
Age (years)	54.53 ± 8.47	54.50 ± 9.83	54.55 ± 7.13	-0.018	0.985
Gender (male, %)	28 (70.0)	18 (90.0)	10 (50.0)	7.619	0.006
BMI (kg/m^2^)	25.36 ± 3.30	25.88 ± 3.65	24.85 ± 2.92	0.980	0.333
Diabetes duration (years)	8.50 (3.25, 10.00)	8.00 (3.00, 10.00)	10.00 (6.00, 10.75)	-1.208	0.227
Hypertension (n, %)	29 (72.5)	14 (70.0)	15 (75.0)	0.125	0.723
SBP (mmHg)	150.65 ± 21.30	145.40 ± 16.89	155.90 ± 24.24	-1.589	0.120
DBP (mmHg)	84.58 ± 11.35	81.75 ± 9.36	87.40 ± 12.65	-1.606	0.117
UACR (ug/mg)	2290.66 (983.56, 4009.99)	1515.67 (775.41, 3065.84)	3152.75 (2507.77, 6197.09)	-2.858	0.004
24hUpro (g/24h)	2.54 (1.56, 6.19)	2.02 (1.41, 2.67)	5.66 (2.54, 7.88)	-3.709	0.001
BUN (mmol/L)	7.75 (6.15, 10.99)	6.79 (5.51, 7.86)	10.40 (7.36, 13.31)	-3.259	0.001
SUA (umol/L)	412.00 (300.25, 457)	391.50 (296.50, 469.50)	426.00 (308.00, 457.00)	-0.379	0.705
Scr (umol/L)	96.60 (78.58, 125.75)	82.60 (66.23, 109.2)	115.90 (85.25, 151.63)	-2.394	0.017
eGFR (mL/min/1.73m^2^)	70.57 ± 28.86	84.93 ± 24.08	56.22 ± 26.38	3.595	0.001
HbA1c (%)	8.43 (6.70, 9.78)	8.52 (7.40, 9.95)	8.15 (6.44, 8.84)	-1.328	0.184
FBG (mmol/L)	7.55 (5.83, 9.95)	8.19 (6.06, 11.79)	6.99 (5.76, 8.75)	-1.403	0.161
Hb (g/L)	125.73 ± 26.34	142.85 ± 22.35	108.60 ± 17.52	5.394	0.000
Alb (g/L)	34.86 ± 6.00	38.65 ± 4.029	31.07 ± 5.23	5.132	0.000
Fib (g/L)	4.13 ± 0.94	3.75 ± 1.01	4.52 ± 0.70	-2.812	0.008
TC (mmol/L)	5.08 (4.03, 6.39)	4.49 (3.47, 5.67)	5.69 (4.87, 6.93)	-2.543	0.011
TG (mmol/L)	1.65 (1.27, 2.3)	1.92 (1.47, 2.37)	1.44 (1.17, 2.47)	-1.170	0.242
HDL-C (mmol/L)	1.25 (1.05, 1.48)	1.07 (0.88, 1.36)	1.29 (1.18, 1.84)	-2.246	0.025
LDL-C (mmol/L)	3.05 (2.12, 3.77)	2.72 (1.74, 4.13)	3.04 (2.12, 3.84)	-1.732	0.083
IFTA (%)	10 (10, 25)	10 (5, 10)	15 (10, 29)	-3.267	0.001
Degree of inflammation (%)	5 (5, 10)	5 (5, 5)	5 (5, 14)	-2.091	0.037

24hUpro, 24-hour urinary protein; Alb, albumin; BMI, body mass index; BUN, blood urea nitrogen; DBP, diastolic blood pressure; eGFR, estimated glomerular filtration rate; FBG, fasting blood glucose; Fib, fibrinogen; Hb, hemoglobin; HbA1c, glycated hemoglobin; HDL-C, high-density lipoprotein cholesterol; IFTA, interstitial fibrosis and renal tubular atrophy; LDL-C, low-density lipoprotein cholesterol; SBP, systolic blood pressure; Scr, serum creatinine; SUA, serum uric acid; TC, total cholesterol; TG, triglycerides; UACR, urinary albumin/creatinine ratio.

### 3.2 Renal pathological changes in control group and DN patients

**Figure 1:**
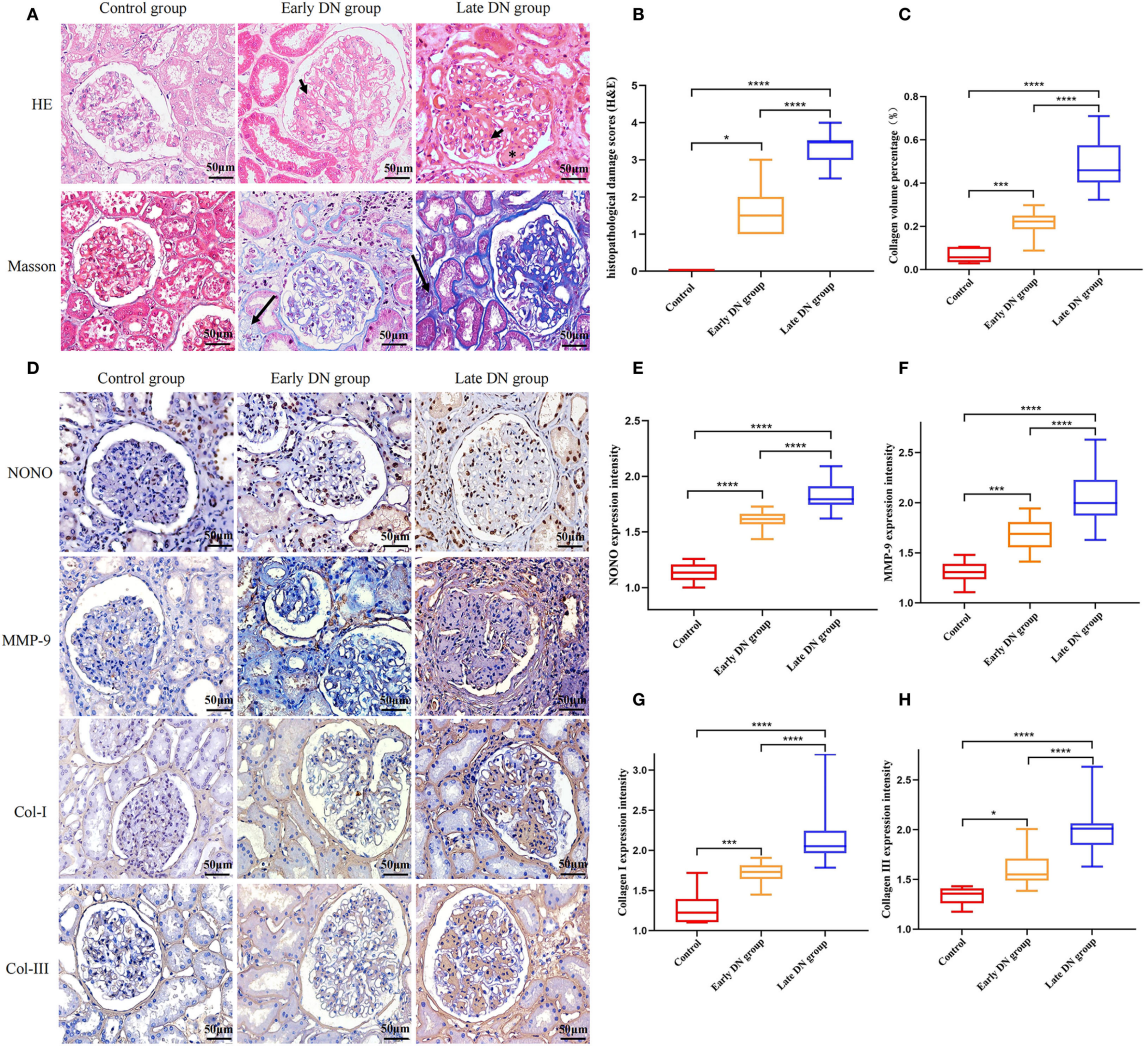
**(A)** HE and Masson staining of renal tissue in each group (×400). Mesangial cell proliferation (arrowheads). Kimmelstiel-Wilson lesions (asterisks). collagen fiber deposition in the renal interstitium (long arrow) **(B)** Semi-quantitative comparison of HE staining results among different groups. **(C)** Quantitative comparison of Masson staining results among different groups. **(D)** Immunohistochemical staining of NONO, MMP-9, Col-I, and Col-III in each group (×400). **(E-H)** Comparison of expression levels of NONO, MMP-9, Col-I, and Col-III in renal tissues of each group. *P<0.05;***P<0.001, ****P<0.0001.

HE staining results showed that in the control group, the kidney structure was clear, and the cell morphology was normal, with no obvious mesangial proliferation or other pathological changes. In the early DN group, kidney tissue showed glomerular hypertrophy, mild diffuse proliferation of mesangial cells and matrix, renal tubular damage, and thickening of the glomerular basement membrane. In the late DN group, the renal lesions were more severe than in the early DN group, with severe diffuse proliferation of glomeruli and matrix, and typical pathological manifestations such as segmental glomerulosclerosis, microangiomas, and K-W nodules (**Figure 1A**). Semi-quantitative analysis of pathological damage revealed that the degree of pathological injury in the early DN group was significantly more severe compared to the control group (*P*<0.05). Furthermore, the extent of renal injury in the late DN group was markedly greater than that in the early DN group, with a statistically significant difference (*P*<0.05) (**Figure 1B**).

Masson staining results showed a small amount of blue collagen deposition in the renal interstitium of the control group, with no obvious interstitial fibrosis. Compared with the control group, both the early and late DN groups showed a large amount of collagen fiber deposition in the renal interstitium, with the late DN group exhibiting more significant changes than the early DN group (**Figure 1A**). Further semi-quantitative analysis of the Masson staining results using Image J software showed an increase in the collagen volume percentage compared to the control group, with statistical significance (*P*<0.05). The late DN group also exhibited a higher collagen volume percentage than the early DN group, and the difference was statistically significant (*P*<0.05) (**Figure 1C**).

Immunohistochemical staining results showed that in the control group, NONO was expressed in the nuclei of some renal tubular epithelial cells, mesangial cells of the glomeruli, and interstitial cells, appearing brownish yellow or dark brown under the microscope. MMP-9 was expressed in some mesangial cells of the glomeruli, renal tubular epithelial cells, and renal interstitium. Col-I and Col-III were mainly expressed in mesangial cells of the glomeruli and renal interstitium, showing brownish yellow cytoplasm with blue cell nuclei. Compared with the control group, the expression of NONO, MMP-9, Col-I, and Col-III was increased in both the early and late DN groups, with more pronounced expression in the late DN group (**Figure 1D**). Additionally, further semi-quantitative analysis of the protein expression levels of NONO, MMP-9, Col-I, and Col-III revealed that their expression levels were higher in both the early and late DN groups compared to the control group (*P*<0.05), with the late DN group showing higher expression levels than the early DN group (*P*<0.05) (**Figure 1E-H**).

### 3.3 Immunofluorescence of NONO and WT1 or AQP1 in the kidneys

**Figure 2:**
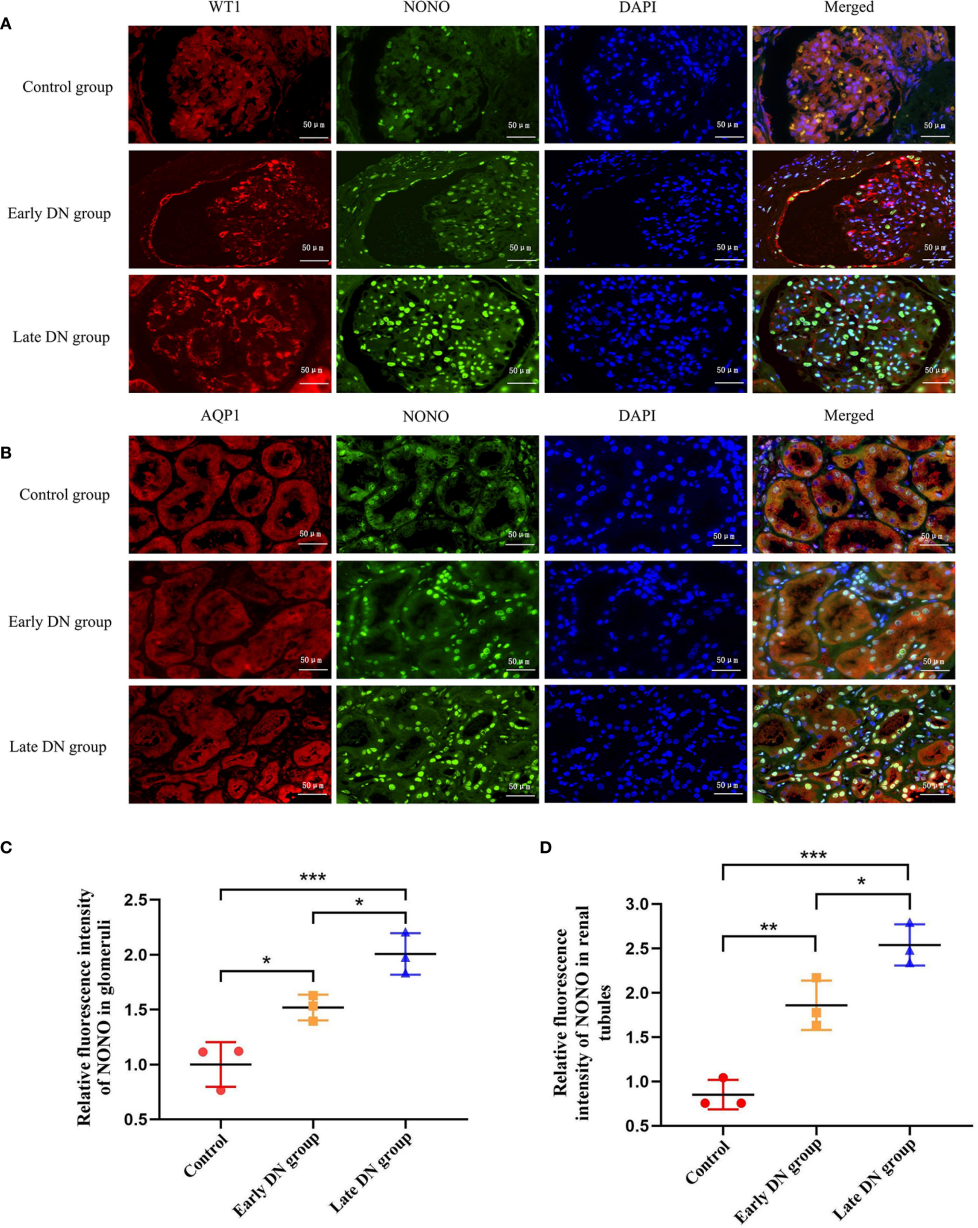
**(A)** Immunofluorescence staining was used to detect the expression levels and co-localization of WT-1 (red) and NONO (green) in glomeruli of each group (×400). **(B)** Immunofluorescence staining was used to detect the expression levels and co-localization of AQP1 (red) and NONO (green) in renal tubules of each group (×400). **(C, D)** Quantitative analysis of average fluorescence intensity of NONO in glomeruli and tubules of renal tissues in each group. *P<0.05;**P<0.01;***P<0.001.

Wilms’ Tumor 1 (WT-1) is specifically expressed in podocytes in glomerular tissue and can be used as a biomarker for glomerular localization, while Aquaporin 1 (AQP1) is specifically expressed in proximal convoluted tubules and can be used as a biomarker for tubular localization (19). By using immunofluorescence, we conducted double staining for NONO with WT1 or Aquaporin 1 in renal tissue. The results showed that in glomerular tissue, NONO fluorescence (green) could be observed within the WT1 fluorescence (red) region (**Figure 2A**). In renal tubules, NONO fluorescence (green) was seen within the AQP1 fluorescence (red) region (**Figure 2B**), confirming the expression of NONO in both glomeruli and renal tubules.

Further quantitative analysis of the immunofluorescence results showed that compared with the control group, the expression of NONO in renal tissue was higher in both the early and late DN groups (*P*<0.05). The expression in the late DN group was significantly higher than in the early DN group (*P*<0.05) (**Figures 2C, D**).

### 3.4 Correlation between NONO and MMP-9 expression levels and clinical pathological indicators

A multiple linear regression model was constructed with the immunohistochemical detection of NONO expression in renal tissue as the dependent variable and other clinical and pathological indicators as independent variables. Correlation analysis revealed that NONO expression levels were positively correlated with clinical indicators such as SBP, DBP, UACR, 24hUpro, BUN, Fib, TC, and HDL-C (*r* values were 0.335, 0.343, 0.367, 0.468, 0.479, 0.322, 0.358, and 0.376, respectively, *P*<0.05), and negatively correlated with Hb and Alb (*r* values were -0.529 and -0.435, respectively, *P*<0.05). NONO expression was also positively correlated with the expression levels of MMP-9, Col-I, Col-III, collagen volume percentage, IFTA involvement ratio, and degree of inflammatory cell infiltration (*r* values were 0.552, 0.623, 0.490, 0.648, 0.418, and 0.341, respectively, *P*<0.05). No significant correlation was found between NONO expression and age, BMI, diabetes duration, SUA, Scr, eGFR, HbA1c, FBG, TG, and LDL-C (*P* > 0.05) (**Table 2**).

**Table 2:** Correlation analysis between the expression levels of NONO and MMP-9 in renal tissue of DN patients and clinical pathological indicators.

Characteristic	NONO expression level		MMP-9 expression level	
	*r*	*P*	*r*	*P*
Age (years)	0.097	0.551	-0.241	0.139
BMI (kg/m^2^)	-0.152	0.348	-0.228	0.164
Diabetes duration (years)	0.265	0.099	0.002	0.989
SBP (mmHg)	0.335	0.035	0.401	0.011
DBP (mmHg)	0.343	0.030	0.344	0.032
UACR (ug/mg)	0.367	0.020	0.380	0.017
24hUpro (g/24h)	0.468	0.003	0.361	0.026
BUN (mmol/L)	0.479	0.002	0.424	0.008
SUA (umol/L)	0.017	0.919	-0.068	0.680
Scr (umol/L)	0.248	0.123	0.387	0.015
eGFR (mL/min/1.73m^2^)	-0.309	0.052	-0.410	0.010
HbA1c (%)	-0.233	0.147	-0.272	0.094
FBG (mmol/L)	-0.121	0.470	-0.101	0.554
Hb (g/L)	-0.529	<0.001	-0.575	<0.001
Alb (g/L)	-0.435	0.005	-0.487	0.002
Fib (g/L)	0.322	0.043	0.024	0.133
TC (mmol/L)	0.358	0.027	0.335	0.043
TG (mmol/L)	-0.133	0.425	-0.145	0.392
HDL-C (mmol/L)	0.376	0.017	0.356	0.026
LDL-C (mmol/L)	0.220	0.173	0.138	0.403
IFTA (%)	0.418	0.008	0.456	0.004
Degree of inflammation (%)	0.341	0.034	0.343	0.035
NONO expression level	–	–	0.552	<0.001
MMP-9 expression level	0.552	<0.001	–	–
Col-I expression level	0.623	<0.001	0.583	<0.001
Col-III expression level	0.490	0.001	0.552	<0.001
Collagen volume percentage (%)	0.648	<0.001	0.658	<0.001

24hUpro, 24-hour urinary protein; Alb, albumin; BMI, body mass index; BUN, blood urea nitrogen; Col-I, type I collagen; Col-III, type III collagen; DBP, diastolic blood pressure; eGFR, estimated glomerular filtration rate; FBG, fasting blood glucose; Fib, fibrinogen; Hb, hemoglobin; HbA1c, glycated hemoglobin; HDL-C, high-density lipoprotein cholesterol; IFTA, interstitial fibrosis and renal tubular atrophy; LDL-C, low-density lipoprotein cholesterol; MMP-9, matrix metalloproteinases-9; NONO, Non-POU domain containing octamer-binding protein; SBP, systolic blood pressure; Scr, serum creatinine; SUA, serum uric acid; TC, total cholesterol; TG, triglycerides; UACR, urinary albumin/creatinine ratio.

A similar correlation analysis was performed for MMP-9 expression, with results showing that MMP-9 expression was positively correlated with clinical indicators such as SBP, DBP, UACR, 24hUpro, BUN, Scr, TC, and HDL-C (*r* values were 0.401, 0.344, 0.380, 0.361, 0.424, 0.387, 0.335, and 0.356, respectively, *P*<0.05), and negatively correlated with eGFR, Alb, and Hb (*r* values were -0.410, -0.487, and -0.575, respectively, *P*<0.05). MMP-9 expression was also positively correlated with NONO, Col-I, Col-III expression levels, collagen volume percentage, IFTA involvement ratio, and degree of inflammatory cell infiltration (*r* values were 0.552, 0.583, 0.552, 0.658, 0.456, and 0.343, respectively, *P*<0.05). No significant correlation was found between MMP-9 expression and age, BMI, diabetes duration, SUA, HbA1c, FBG, Fib, TG, and LDL-C (*P* > 0.05) (**Table 2**).

### 3.5 Multivariate linear regression analysis of NONO influencing factors

A multivariate linear regression analysis was performed using NONO expression in renal tissue as the dependent variable and the indicators that were correlated with NONO expression as independent variables. The final optimal model showed that UACR and Col-I expression levels were independent influencing factors for NONO (F=15.854, *P*<0.001). This indicates that as UACR and Col-I expression levels increase, the expression of NONO also increases (**Table 3**).

**Table 3:** Multiple linear regression analysis of NONO and various indicators.

Characteristic	Unstandardized coefficients		Standardized coefficient	*t*	*P*	Variance inflation factor (VIF)
	*B*	Standard error (S.E)	*Beta*			
UACR (ug/mg)	<0.001	0.000	0.400	3.019	0.005	1.028
Col-I expression level	0.346	0.083	0.554	4.185	<0.001	1.028

Col-I, type I collagen; UACR, urinary albumin/creatinine ratio.

### 3.6 Relationship between renal tissue NONO and prognosis of DN patients

Univariate Cox regression was used to identify risk factors for poor prognosis in DN patients by evaluating clinical and pathological indicators, including gender (coded as: female=0, male=1), hypertension history (coded as: none=0, yes=1), and other continuous variables. The analysis revealed that gender, UACR, 24hUpro, Hb, Alb, TC, HDL-C, IFTA, NONO expression, MMP-9 expression, Col-I expression, Col-III expression, and collagen volume percentage were all factors influencing the prognosis of DN patients (*P*<0.05) (**Table 4**).

**Table 4:** Analysis of risk factors affecting poor prognosis in DN patients.

Characteristic	HR	95%CI	*P*-value
Age (years)	1.00	0.94-1.06	0.959
Gender (male, %)	0.29	0.11-0.82	0.019
BMI (kg/m^2^)	0.91	0.79-1.05	0.180
Diabetes duration (years)	0.97	0.88-1.07	0.592
Hypertension (n, %)	0.79	0.25-2.47	0.678
SBP (mmHg)	1.02	0.99-1.04	0.163
DBP (mmHg)	1.02	0.99-1.07	0.228
UACR (ug/mg)	1.00	1.00-1.00	0.013
24hUpro (g/24h)	1.16	1.04-1.30	0.009
BUN (mmol/L)	1.08	0.99-1.19	0.101
SUA (umol/L)	1.00	1.00-1.01	0.673
Scr (umol/L)	1.00	1.00-1.01	0.341
eGFR (mL/min/1.73m^2^)	0.98	0.97-1.00	0.102
HbA1c (%)	0.82	0.60-1.10	0.187
FBG (mmol/L)	0.92	0.77-1.11	0.392
Hb (g/L)	0.95	0.92-0.98	0.001
Alb (g/L)	0.81	0.73-0.90	<0.001
Fib (g/L)	1.61	0.96-2.7	0.071
TC (mmol/L)	1.36	1.06-1.74	0.016
TG (mmol/L)	0.54	0.24-1.23	0.143
HDL-C (mmol/L)	5.64	1.81-17.63	0.002
LDL-C (mmol/L)	1.26	0.91-1.75	0.171
IFTA (%)	1.08	1.03-1.13	0.003
Collagen volume percentage (%)	1.04	0.97-1.12	0.286
NONO expression level	1.85	1.31-2.61	<0.001
MMP-9 expression level	1.63	1.29-2.07	<0.001
Col-I expression level	1.65	1.31-2.08	<0.001
Col-III expression level	1.61	1.27-2.02	<0.001
Collagen volume percentage (%)	2.25	1.59-3.18	<0.001

24hUpro, 24-hour urinary protein; Alb, albumin; BMI, body mass index; BUN, blood urea nitrogen; Col-I, type I collagen; Col-III, type III collagen; DBP, diastolic blood pressure; eGFR, estimated glomerular filtration rate; FBG, fasting blood glucose; Fib, fibrinogen; Hb, hemoglobin; HbA1c, glycated hemoglobin; HDL-C, high-density lipoprotein cholesterol; IFTA, interstitial fibrosis and renal tubular atrophy; LDL-C, low-density lipoprotein cholesterol; MMP-9, matrix metalloproteinases-9; NONO, Non-POU domain containing octamer-binding protein; SBP, systolic blood pressure; Scr, serum creatinine; SUA, serum uric acid; TC, total cholesterol; TG, triglycerides; UACR, urinary albumin/creatinine ratio.

**Figure 3:**
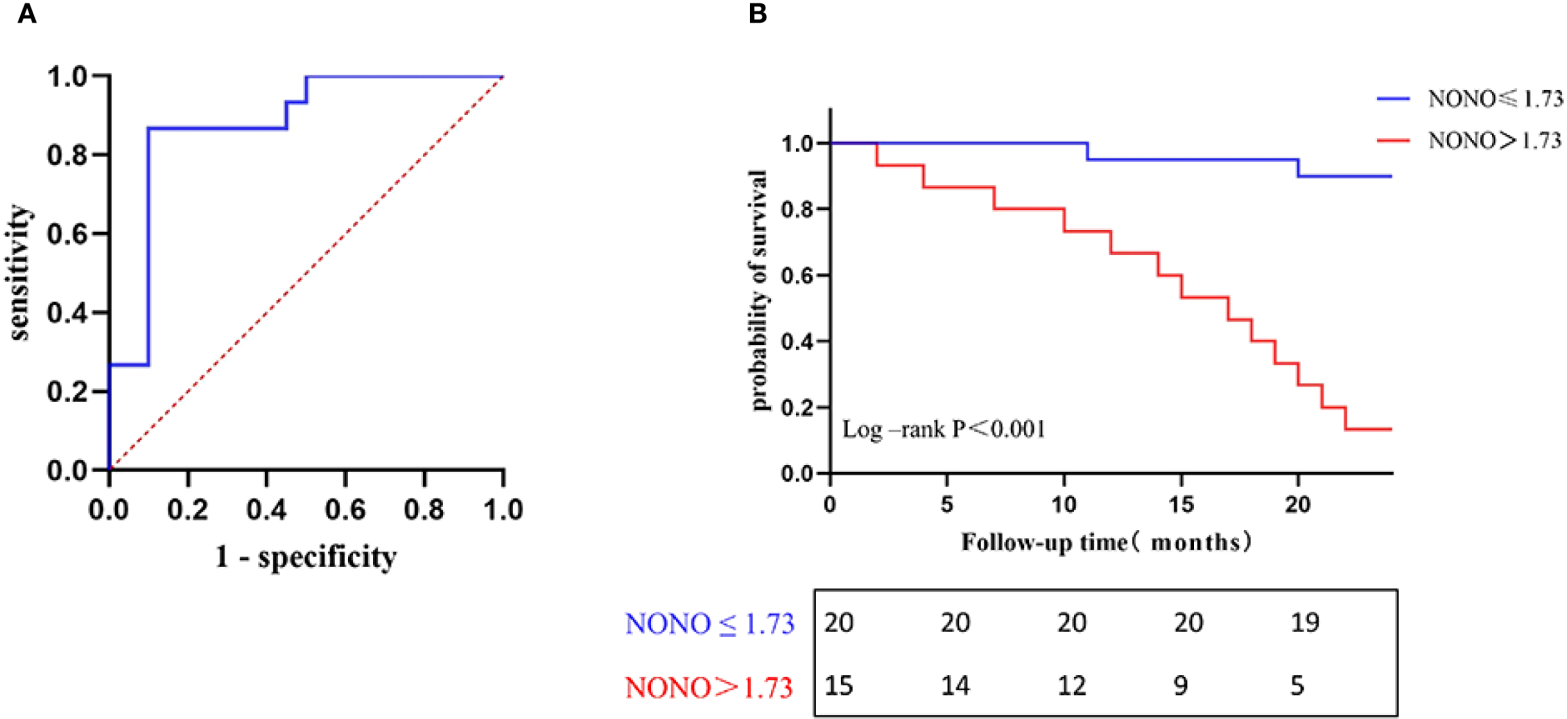
**(A)** ROC curve for predicting poor prognosis of DN. **(B)** Kaplan-Meier survival curves of NONO expression levels associated with renal progression in DN patients.

To study the diagnostic and predictive values of NONO expression for predicting poor prognosis in DN patients, ROC curve analysis was performed. The results showed that the area under the AUC curve for NONO expression in predicting composite renal outcomes was 0.877 (95% CI: 0.756-0.997, *P*<0.05). The optimal cutoff value for NONO expression was 1.734, with a sensitivity of 86.7% and specificity of 90.0% (**Figure 3A**).

To compared kidney progression-free survival in DN patients followed for 24 months, patients were divided into two groups based on the optimal cutoff value of NONO expression (≤1.734 and >1.734). Kaplan-Meier survival analysis showed that the kidney progression-free survival rates at 15, 20, and 24 months were 95.0%, 90.0%, and 90.0% in the NONO ≤ 1.734 group, respectively, while the rates were 53.3%, 26.7%, and 13.3% in the NONO > 1.734 group. Log-Rank test analysis revealed a significant difference in kidney progression-free survival between the two groups (*P*<0.001) (**Figure 3B**).

## 4 Discussion

The results of this study found that NONO is primarily distributed in the mesangial cells of the glomerulus, tubular epithelial cells, and the nuclei of interstitial cells in the kidney. Compared to the control group, the expression level of NONO in the kidney tissue of DN patients was significantly higher, indicating that NONO may be involved in the pathogenesis of DN. When compared with the early-stage DN group, we found that the expression level of NONO was further elevated in the late-stage DN group, suggesting a correlation between NONO and the progression of pathological changes in DN. Through correlation analysis, it was shown that the expression level of NONO was positively correlated with BUN, UACR, and 24hUpro, and negatively correlated with Hb and Alb. Moreover, NONO expression was significantly positively correlated with fibrosis-related pathological indicators such as Col-I, Col-III expression, collagen volume percentage, and the proportion of IFTA. Although our study did not reveal a statistically significant correlation between the expression level of NONO and eGFR (P=0.052), this outcome may have been influenced by the limited sample size, potentially introducing bias into the results. Further multivariate linear regression analysis revealed that UACR and Col-I expression were independently associated with the expression of NONO. BUN, UACR, 24hUpro, Hb, and Alb reflect the degree of kidney function damage and are important indicators for assessing kidney function progression (20–22). Therefore, our results suggest that the expression level of NONO is closely associated with kidney injury and fibrosis. The differential expression of NONO in early and late-stage DN can reflect the degree of kidney damage in DN patients.

A study by Kenji et al. reported that highly expressed NONO could interact with the Sox9 transcription factor to promote chondrocyte differentiation and ECM protein expression, playing an important role in cartilage formation (23). In NONO gene knockout mice, excessive proliferation of fibroblasts and reduced collagen production were observed in skin wounds, hindering wound healing and leading to immature granulation tissue formation (24). However, in studies on mice lacking NONO, it was found that excessive proliferation of cardiac fibroblasts and restricted migration induced collagen generation and deposition, and NONO overexpression could reverse this effect. Furthermore, the team later demonstrated a correlation between NONO deficiency and cardiac defects in mice, suggesting that NONO may influence cardiac structure and fibrosis by mediating ECM metabolism, cell proliferation, and migration (8). These findings indicate that the regulatory role of NONO in ECM is not consistent across different tissues, organs, and disease conditions. In cases of hyperglycemia, hemodynamic and metabolic disorders, RIF serves as a critical factor in the progression of DN, contributing to ECM deposition, structural and functional impairment of the kidney, and ultimately leading to renal failure (25). Thus, we hypothesize that NONO may participate in DN kidney damage and RIF by regulating the ECM process.

The correlation analysis in our study also showed that NONO was positively correlated with SBP, DBP, and Fib, suggesting that NONO may have some correlation with blood pressure levels and coagulation functions. Additionally, NONO was positively correlated with TC and HDL-C, indicating that NONO may be involved in the transcriptional regulation of lipid metabolism, which is consistent with the findings of Benegiamo et al (26). This study found that in mice lacking NONO, fatty acid oxidation replaced glucose storage, leading to impaired glucose tolerance and reduced glycogen and blood lipids. The specific mechanism involves NONO’s transcriptional regulation of genes related to glucose and lipid metabolism, contributing to the development of metabolic diseases.

Finally, we predicted the composite kidney outcomes in DN patients based on NONO expression levels in kidney tissue using the ROC curve, selecting the optimal cutoff value of 1.734. The sensitivity of this prediction for adverse DN prognosis was 86.7%, and the specificity was 90.0%. The Kaplan-Meier survival analysis showed that patients with high NONO expression in kidney tissue had a lower 24-month kidney progression-free survival rate. This suggests that the expression level of NONO in kidney tissue is a good predictor of DN progression, which may be related to NONO’s involvement in the RIF process.

This study also found that MMP-9 is primarily expressed in mesangial cells of the glomerulus, tubular epithelial cells, and renal interstitial cells. Compared to the control group, the expression level of MMP-9 was significantly elevated in DN patient kidney tissue, and its expression was more pronounced in late-stage DN patients. The correlation analysis showed that MMP-9 expression levels were positively correlated with kidney function and injury-related indicators, including Scr, UACR, 24hUpro, and BUN, and negatively correlated with eGFR, Alb, and Hb. These results suggest that MMP-9 expression is closely related to kidney damage. Additionally, we found that MMP-9 expression was positively correlated with fibrosis-related indicators such as Col-I expression, Col-III expression, collagen volume percentage, and the proportion of IFTA. Several studies have reported that MMP-9 is a gelatinase that degrades ECM and participates in the signaling regulation of various cytokines, accelerating the progression of kidney fibrosis (27, 28). Therefore, our results suggest that MMP-9 may participate in RIF, and its underlying mechanism may also be related to ECM deposition. This is consistent with previous reports. Li et al. found that DM can upregulate MMP-9 activity and expression in mouse glomeruli, while MMP-9-deficient mice showed reduced glomerular hyperfiltration and proteinuria levels, promoting kidney size and function recovery (29). Wang et al. reported that MMP-9 promotes neutrophil infiltration by increasing inflammation, macrophage aggregation, and kidney fibrosis in a mouse model of unilateral ureteral obstruction (UUO), and inhibiting MMP-9 expression in early acute kidney injury can delay the progression of kidney fibrosis (30). Knockout of the MMP-9 gene reduced the activation of myofibroblasts and EMT in UUO mice, thereby reducing ECM deposition and kidney tubular basement membrane damage, significantly improving RIF (31).

To further explore the relationship between NONO and MMP-9, we conducted correlation analysis and found that NONO expression levels were significantly positively correlated with MMP-9 expression in DN patients. Previous studies have shown that NONO can activate NF-κB and upregulate MMP-9 expression, participating in ECM metabolism (10). In studies on the abdominal aorta of mice, knockout of the NONO gene downregulated the expression of MMP-2 and MMP-9, reducing collagen degradation (9). Our findings are consistent with these reports and suggest a close relationship between NONO and MMP-9 in ECM metabolism. Therefore, this study is the first to explore the potential role of NONO in RIF, proposing that NONO may regulate MMP-9 expression to promote ECM deposition, ultimately exacerbating DN kidney fibrosis and the severity of the disease. Its expression level has a good predictive ability for the progression of DN. However, the current study had a relatively small sample size, which necessitates future research with expanded cohorts to validate the experimental findings. Furthermore, this investigation represented only a preliminary exploration in patients with DN and did not fully elucidate the precise mechanism by which NONO regulates renal fibrosis. In addition, the potential regulatory role of NONO in MMP-9 expression during the fibrotic process, as well as its specific functional and molecular mechanisms, remains unclear. Therefore, more comprehensive studies utilizing both cellular and animal models are required to further investigate these aspects.

## 5 Conclusions

In conclusion, this study suggests that NONO is highly expressed in the kidney tissue of DN patients. The expression levels of NONO and MMP-9 in the kidney tissue of DN patients are positively correlated with Col-I, Col-III expression, collagen volume percentage, and kidney injury-related clinical indicators. NONO expression is also positively correlated with MMP-9 expression in DN kidney tissue. High expression of NONO is associated with poor renal prognosis in DN patients. The study suggests that NONO in kidney tissue may participate in the fibrosis of DN kidney tissue by regulating MMP-9 levels.

## Data Availability

The raw data supporting the conclusions of this article will be made available by the authors, without undue reservation.
